# P-743. Correlating Climate Conditions with *Pseudomonas aeruginosa* Prevalence in Diabetic Foot Infections within the United States

**DOI:** 10.1093/ofid/ofae631.939

**Published:** 2025-01-29

**Authors:** Rebecca Winski, Kari A Mergenhagen, Arthur Chan, Jiachen Xu, Bethany A Wattengel

**Affiliations:** VA WNY Healthcare System, Buffalo, New York; VA WNY Healthcare System, Buffalo, New York; Veterans Affairs Western New York Healthcare System, Buffalo, New York; Veterans Affairs Western New York Healthcare System, Buffalo, New York; VA WNY Healthcare System, Buffalo, New York

## Abstract

**Background:**

Diabetes-related foot infections (DFIs) are often difficult to treat and can result in amputation or even death if allowed to progress. The 2023 ‘International Working Group on the Diabetic Foot (IWGDF)/Infectious Disease Society of America (IDSA) Guidelines on the Diagnosis and Treatment of Diabetes-Related Foot Infections’ provides recommendations on which pathogens to target with empiric antibiotic therapy depending on which solar climate region a patient is from. The United States (US) has numerous different climate zones that can be classified using the US Department of Energy International Energy Conservation Code (IECC), which utilizes annual temperature and precipitation data to assign a climate designation to each county within the US.

IECC Climate Map

**Figure:**
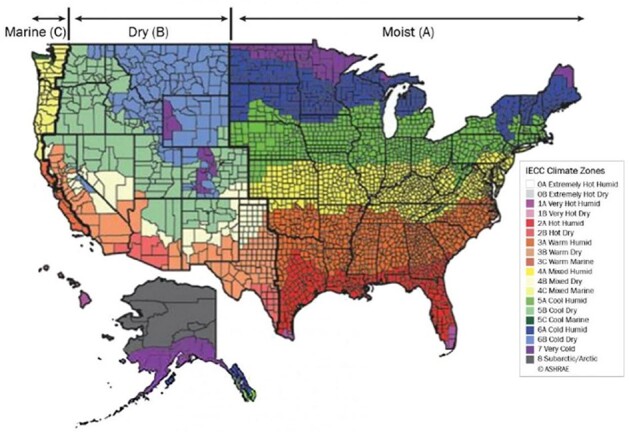

**Methods:**

This study was a retrospective national cohort study of all Veterans aged 18 years of age or older between 1/1/2010 – 3/23/2024 with diabetes mellitus and a cultured lower extremity wound. National Veteran data was obtained from the VA Corporate Data Warehouse (CDW) via Structured Query Language (SQL) Server Management Studio. SQL coding was used to acquire demographic information, ICD diagnoses, lower extremity culture results, and Charlson Comorbidity Index for all patients who met inclusion criteria.

Pseudomonas Prevalence

**Figure:**
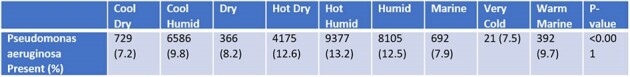

**Results:**

The prevalence of Pseudomonas significantly varied between US climates. Pseudomonas was most prevalent within the Hot Humid climate, where it was isolated in 13.2% of DFI cultures. Pseudomonas was least prevalent within the Cool Dry climate, where it was only isolated in 7.2% of cultures.

**Conclusion:**

The prevalence of DFI organisms varies within different US climates. Utilization of local climate information may allow for more accurate and targeted empiric antibiotic selection when treating DFIs.

**Disclosures:**

**All Authors**: No reported disclosures

